# No Major Role for Insulin-Degrading Enzyme in Antigen Presentation by MHC Molecules

**DOI:** 10.1371/journal.pone.0088365

**Published:** 2014-02-07

**Authors:** Slobodan Culina, François-Xavier Mauvais, Hsiang-Ting Hsu, Anne Burgevin, Suzanne Guénette, Anna Moser, Peter van Endert

**Affiliations:** 1 Institut National de la Santé et de la Recherche Médicale, Unité 1013, Paris, France; 2 Université Paris Descartes, Sorbonne Paris Cité, Faculté de Médecine Paris Descartes, Paris, France; 3 Genetics and Aging Research Unit, MassGeneral Institute for Neurodegenerative Disease, Charlestown, Massachusetts, United States of America; Centre de Recherche Public de la Santé (CRP-Santé), Luxembourg

## Abstract

Antigen presentation by MHC class I molecules requires degradation of epitope source proteins in the cytosol. Although the preeminent role of the proteasome is clearly established, evidence suggesting a significant role for proteasome-independent generation of class I ligands has been reported repeatedly. However, an enzyme responsible for such a role has not been identified. Recently insulin-degrading enzyme (IDE) was shown to produce an antigenic peptide derived from the tumor antigen MAGE-A3 in an entirely proteasome-independent manner, raising the question of the global impact of IDE in MHC class I antigen processing. Here we report that IDE knockdown in human cell lines, or knockout in two different mouse strains, has no effect on cell surface expression of various MHC class I molecules, including allomorphs such as HLA-A3 and HLA-B27 suggested to be loaded in an at least a partly proteasome-independent manner. Moreover, reduced or absent IDE expression does not affect presentation of five epitopes including epitopes derived from beta amyloid and proinsulin, two preferred IDE substrates. Thus, IDE does not play a major role in MHC class I antigen processing, confirming the dominant and almost exclusive role of the proteasome in cytosolic production of MHC class I ligands.

## Introduction

The principal task of MHC class I (MHC-I) molecules is to present breakdown products of proteins synthesized by the presenting cell. The proteases involved in production of MHC-I ligands have been characterized in detail [1]. In the vast majority of cases, cytosolic proteasome complexes initiate degradation of the source proteins, producing peptides with a length of about 5 to 20 amino acids. These can be shortened by various aminopeptidases in the cytosol which in some cases have been shown to be involved in production of antigenic peptides, although the net effect tends to be a loss in class I ligands [2]. Endoplasmic reticulum aminopeptidases play a more important role in epitope production [3], while carboxypeptidases residing in the cytosol and the endoplasmic reticulum have only recently been described to trim some MHC-I ligands [4], [5].

Although the dominant role of the proteasome is well documented and widely recognized, observations suggestive of alternative proteases responsible for the initial attack of source proteins yielding class I ligands have been known for a long time [6]. Among these, the long-term survival of cell lines in the presence of proteasome inhibitors was initially interpreted as evidence for a back-up protease but then turned out to reflect incomplete proteasome inhibition [7]. The fact that cell surface expression of some MHC-I allomorphs is not affected, or even increased, in the presence of proteasome inhibitors, might also be due to partial inhibition, although some studies observed the phenomenon when the relevant proteasome subunits were inhibited at 70 to 80 percent [8], [9]. MHC-I allomorphs preferring ligands with a carboxyterminal lysine such as HLA-A3 appeared to be especially “proteasome independent” although peptides with hydrophobic residues in this position could also be eluted from proteasome-inhibited HLA-A3-expressing cells [8], [10]. Another interesting case is HLA-B27; according to a recent report, about 20 to 30 percent of its ligands appear proteasome-independent and are derived from small basic proteins [11]. Thus, a significant contribution of proteases other than the proteasome to initial antigen degradation in the cytosolic MHC-I processing pathway cannot be ruled out.

The first protease suggested to produce proteasome-independent ligands was tripeptidyl peptidase II (TPPII), a large cytosolic aminopeptidase [12]. Due to its (weak) trypsin-like endoprotease activity, TPPII was a candidate for production of peptides with basic carboxyterminal residues [13]. However, although the enzyme could be shown to contribute to production of some peptides [14], [15], analysis of TPPII-deficient mice produced by several groups did not support a more general role in antigen processing [16], [17]. Moreover, degradation of a complete antigenic protein by TPPII *in vitro* has never been demonstrated.

More recently, the group of B. van den Eynde in collaboration with us has shown that IDE can produce an antigenic peptide derived from the tumor antigen MAGE-A3 in a proteasome-independent manner [18]. IDE is a ubiquitous enzyme with a predominant cytosolic location that degrades preferentially small substrates such as insulin or glucagon although oxidized hemoglobin can also be broken down by it [19]. IDE is unusual in that it seems to select structural features of substrates rather than specific sequences. Thus it has been proposed that IDE prefers substrates with a tendency to form amyloids, consistent with its capacity to degrade amyloid beta, shared by few proteases [20]. Given that amyloid formation generally is a type of misfolding, and that current evidence suggests that many source proteins for class I ligands correspond to defective and therefore possibly misfolded proteins [21], it was conceivable that IDE plays a larger role in MHC-I antigen processing. In this study we examined the effect of IDE knockdown or knockout on global MHC-I expression as well as presentation of a variety of antigens. Our results demonstrate that IDE does not play a more general role in peptide supply to MHC molecules.

## Materials and Methods

### Cell Lines

Human cervix carcinoma (HeLa) and colon carcinoma (HCT116) cell lines were purchased from ATCC. A human embryonic kidney (HEK293) cell line was kindly provided by G. de Saint Basile, Paris; H-2D^d^ transfected HeLa cells by M. delVal, Madrid [22]; H-2K^b^ transfected HeLa cells by I. York, Worcester, MA [17]; HLA-A2 and B27-transfected HeLa cells by J. Lopez de Castro, Madrid (unpublished); H-2K^d^-transfected HEK293 cells expressing a fusion between islet-specific glucose-6-phosphate catalytic subunit related protein (IGRP) and GFP by P. Santamaria, Calgary [23]; the mouse thymoma EL4 cell line (H-2^b^) and an EL4 clone transfected with ovalbumin (EG7) were kindly provided by E. Tartour, Paris. All cell lines were cultured in DMEM media supplemented by heat-inactivated FBS, 100 U/ml penicillin, 100 µg/ml streptomycin and 1% non-essential amino acids.

### siRNA Transfection

Cell lines were transfected with an siRNA set of 4 *Smart pool* heteroduplexes (Thermo Scientific ) specific for human IDE (siIDE, duplex 1∶5′ UCA AAG GGC UGG GUU AAU AUU 3′, 5′ UAU UAA CCC AGC CCU UUG AUU 3′; duplex 2∶5′ ACA CUG AGG UUG CAU AUU UUU 3′, 5′ AAA UAU GCA ACC UCA GUG UUU 3′; duplex 3∶5′ GAA CAA AGA AAU ACC CUA AUU 3′, 5′ UUA GGG UAU UUC UUU GUU CUU 3′; duplex 4∶5′ GAA GUU ACG UGC AGA AGG AUU 3′, 5′ UCC UUC UGC ACG UAA CUU CUU 3′). 5×10^6^ cells were re-suspended in 250 µl of transfection buffer (PBS, 10 mM HEPES pH 8.2) and transfected with a final concentration of 100 nM or 400 nM of each duplex by electroporation (250 V, 960 µF). A non-targeting siRNA duplex pool (siNTG) was used as control. Twenty-four hours, 36 h, 48 h and 72 h after transfection, knockdown efficiency was verified by immunoblotting, reverse transcriptase-PCR (RT-PCR) and fluorescence microscopy.

### RNA Isolation, cDNA Synthesis and Real-time Quantitative RT-PCR

Total RNA was extracted from 2×10^6^ siRNA-transfected cells with the QuickPrep™ extraction kit (GE Healthcare). Total RNA was reverse transcribed into cDNA using the ImProm-II™ Reverse Transcription System (Promega). Quantitative real-time PCR was performed and quantified with a Prism 7700 System (ABI/Life Technologies) using the SYBR Green master mix. The conditions for PCR were as follows: 1 cycle 50°C for 2 min, 1 cycle 95°C for 5 min, and 40 cycles 95°C for 30 s, 55°C for 30 s and 68°C for 1 min. The Ct values obtained were normalized to GAPDH and Actin B expression. The following primer sequences were used: IDE forward 5′-AAA AAG AGG CGA CAC CAT ACC -3′, IDE reverse 5′- AGG TAC AAA TAG GCC ATG TT -3′, GAPDH forward 5′-TGC ACC ACC AAC TGC TTA GC-3′, GAPDH reverse 5′- GGC ATG GAC TGT GGT CAT GAG-3′, Actin B forward 5′- CTG GAA CGG TGA AGG TGA CA-3′ and Actin B reverse 5′- AAG GGA CTT CCT GTA ACA ATG-3′.

### Western Blotting

siRNA transfected cells were lysed in Laemmli buffer, denatured by boiling for 15 min, separated on a 10% SDS-PAGE gel and transferred onto polyvinylidene fluoride membranes (GE Healthcare). Membranes were blocked using non-fat dry milk in Tris-buffered saline with 0.1% Tween 20 overnight at 4°C and probed with an IDE-specific monoclonal antibody (mAb; clone 9B12, 0.5 µg/mL; Covance) and an actin B-specific (1∶5000, Sigma) mAb as loading control. Staining was visualized using HRP-conjugated secondary Abs (1∶25,000; Jackson ImmunoResearch), an ECL substrate (Immobilon™, Millipore) and a Fuji LAS-1000 CCD camera.

### Immunofluorescence

Three days after siRNA transfection, 1×10^5^ HeLa cells were cultured on 0.033% poly-L-lysine-treated 12 mm glass coverslips. Cells were fixed with 4% paraformaldehyde in PBS and quenched with 100 mM glycine, washed in PBS, treated with 0.2% BSA, 0.05% saponine (Sigma) and incubated for 45 min with 5 µg/ml of anti-IDE mAb. After washing, cells were incubated for 45 min with 10 µg/ml FITC-labeled goat anti-mouse antibodies (Abs; Jackson), washed again, fixed with 2% paraformaldehyde followed by glycine quenching and mounted with Fluoprep (BioMérieux). Images were taken with a DMI 6000B fluorescence microscope (Leica, Rueil-Malmaison, France) equipped with a 63x PlanApo objective and analyzed by 3D deconvolution using Metamorph®6.2 software (Universal Imaging Corp., Downington, PA).

### Flow Cytometry

Twenty-four hours after siRNA transfection, cells were plated in complete DMEM in 6 well plates with or without 400 U/ml IFN-γ (R&D Systems) at 9×10^5^ or 6×10^5^ cells per well, respectively. Two days later, cells were washed with FACS buffer (PBS, 1.5% BSA, 0.05% NaN_3_), incubated with mAb W6/32 for 1 h at 4°C, washed and stained with FITC-labeled goat anti-mouse Abs (Jackson). Samples were acquired on a FACSCalibur™ (BD Bioscience) and analyzed using FlowJo™ software. Dead cells were excluded by propidium iodide staining.

Expression of MHC molecules was also evaluated on splenocytes of previously described IDE-deficient mice on both C57BL/6 and non-obese diabetic (NOD) backgrounds; the latter mice were produced by back-crossing the published strain [19] ten times to NOD mice (AM and PVE, manuscript in preparation). Cells were stained with anti-CD19/PE (clone 6D5; Biolegend), anti-CD11c/eFluor450 (clone N418; eBioscience), anti-CD11b/PE-Cy (clone M1/70; BD Biosciences), anti-TCR-β/APC (clone H57-597; eBioscience), anti-F4-80/APC-Cy7 (clone RM8; Biolegend), anti-H2-K^b^/biotin (clone AF6-88.5; Biolegend), anti-H2-K^d^/biotin (clone SF1-1.1; Biolegend) or anti-I-A^k^/biotin (cross-reacting with I-A^g7^; clone 10-3.6; Biolegend) or rat anti-mouse IgG2a (Biolegend) Abs followed by streptavidin/PE-CF594 (BD Biosciences). Prior to analysis on a FACSFortessa or a FACSCanto II flow cytometer, cells were stained with 7-AAD (BD Biosciences). At least 3 independent experiments with 2–3 mice per group were performed.

### Flow Cytometry on Acid-stripped Cells

Sixty-five hours after transfection of HEK293 or HeLa B27 cells with siRNA, 1 µM epoxomicin or 10 µg/ml brefeldin A (both from Sigma) was added to the cells for 2 h or 30 min, respectively. Drug treatment was followed by acid removal of surface class I molecules, using a 90 s incubation in cold citrate buffer (pH = 3) for HEK293 cells, and a 120 s incubation in cold glycine buffer (pH = 2.8) for HeLa B27 cells, followed by neutralization with five volumes of DMEM without FBS. The stripped cells were split in two parts, one of which was stained immediately for HLA class I expression, while the other was suspended in DMEM with 10% FBS and incubated for 6 h in the presence of the drugs used before stripping, or without drugs. Finally the cells were stained for expression of HLA-A,B,C (mAb W6/32), HLA A3 (mAb GAP.A3) or HLA-B27 (mAb ME-1), as described above. Alternatively, splenocytes from C57BL/6 mice or NOD IDE wild type (wt) or knockout (ko) mice were treated with cold glycine buffer (pH = 2.8) for 90 s before neutralization by successive washes with five volumes of IMDM without FBS. The acid-stripped cells were either immediately stained for MHC-I expression, or suspended in IMDM with 10% FBS and incubated at 37°C for several periods. Finally the cells were stained for expression of H-2K^b^ (mAb AF6.88-5) or H-2K^d^ (mAb SF1-1.1) in combination with anti-CD19 and anti-CD11c as described above.

### Antigen Presentation Assays

#### Vaccinia-ovalbumin

3×10^6^ HeLa-K^b^ cells were transfected with siRNA. Three days later, the cells were infected with vaccinia viruses expressing ovalbumin (OVA) or peptide SIINFEKL (S8L) for 2 h at a multiplicity of infection (MOI) of 30 and 10, respectively. Then the cells were fixed with 1% formaldehyde, washed successively with 0.2 M glycine pH7.4, pH 7.0 and PBS, and added to T cell receptor (TCR)-transgenic OT-I T cells [24] seeded at 2×10^5^/well in 96 well plates so that an effector:target (E:T) ratio of 2∶1 and 4∶1 resulted. HeLa cells not expressing H-2K^b^ and HeLa-K^b^ cells pulsed for 2 h with 10^−10^M S8L peptide were negative and positive controls, respectively. IFN-γ secretion by OT-I cells was analyzed after a 12 h incubation by standard ELISA. Capture anti-IFN-γ mAb was clone R4-6A_2_ and detection mAb was clone XMG1.2 (both BD Pharmingen), both used at 0.2 µg/50 µl/well. Incubation times were 5 h for supernatant, 45 min for detection mAb, and 30 min for streptavidin-HRP (Sigma). Tetramethylbenzidine was used as substrate. Alternatively 400,000 C57BL/6 IDE wt or ko Mouse Embryonic Fibroblasts (MEFs) or Bone Marrow-derived Dendritic Cells (BM-DCs), prepared as described elsewhere [25] were infected with a vaccinia virus expressing OVA or the control strain WR1354 (obtained from ATCC) at an MOI of 30 and 10 for 2 h or 6 h. H-2K^b^-S8L complexes were detected by sequential incubation with mAb 25D1.16 (10 µg/mL), FITC-labeled goat anti-mouse Abs (1∶50; Biolegend), and Alexa488-labeled goat anti-FITC Abs (1∶100; Invitrogen) and the fluorescence analyzed on a BD FACSCANTO-II analyzer.

#### Vaccinia-gp160

2×10^6^ HeLa-D^d^ cells were transfected with siRNA and infected with vaccinia viruses expressing the HIV envelope glycoprotein protein (gp160) or wt vaccinia virus at an MOI of 30, as described for vaccinia-OVA. Six hours after infection, the cells were added to a previously described CD8+ T cell clone recognizing the epitope RGPGRAVTI at an E:T ratio of 2∶ 1 (1×10^5^ CTLs/well in a 96-well plate). HeLa-D^d^ cells pulsed with 10^−7^M peptide G9I for 2 h, and cells transfected 24 h before addition of CTL with plasmid pMACS4-IRES.II encoding G9I, were used as controls. Cell lysis was assessed using the CytoTox-One™ assay (Promega), which measures release of lactate dehydrogenase by permeable dying cells. The percentage of specific lysis was calculated as: (experimental result – medium background) divided by (maximum lysis – medium background) multiplied with 100.

#### IGRP-GFP

3×10^6^ HEK293-K^d^ cells stably expressing an IGRP-GFP fusion protein were transfected with siRNA. Two days later, the cells were acid-stripped for 120 s with citrate buffer (pH = 2.8) followed by an overnight incubation in DMEM 10% FBS in the presence or absence of 0.1 µM epoxomicin. P815 cells pulsed for 2 h with 10^−6^M superagonist peptide NRP-V7 were used as positive controls. HEK293 and P815 cells were added to TCR-transgenic 8.3 cells recognizing epitope IGRP_206-14_ for 12 h at an E:T ratio of 2∶1 before quantification of IFN-γ secretion by ELISA as described above. 8.3 effector cells were prepared by stimulation of splenocytes from 8.3 mice with irradiated splenocytes from NOD mice and 10^−6^M peptide IGRP_206-14_, and used 5 or 6 days after stimulation.

#### Amyloid-beta and proinsulin-S8L

Amyloid beta and proinsulin expression plasmids were constructed in the vector pTRE-Tight (*Clontech*) containing the Tet Response Element. Initially the pTRE-Tight cloning site was modified by insertion between the SalI and XbaI sites of complementary oligonucleotides encoding the OVA epitope S8L. Then a cDNA encoding proinsulin was amplified from a previously described cloned human preproinsulin sequence [26], using primers containing BamHI (5′) and SalI (3′) sites, and subcloned into the modified vector. Sequences encoding human beta amyloid 1–42 preceded by the signal peptide from the rat Ig kappa-chain or not were synthesized by GeneArt (Munich, Germany) and subcloned as NcoI/SalI fragments into modified pTRE-Tight. HeLa Tet-on cells (Clontech) stably transfected with H-2K^b^ (Hsu et al., submitted) were nucleofected with 4×100 nM siRNA. One day later, the cells were electroporated with 20 µg of the described pTRE-Tight plasmids. Expression was induced immediately by addition of 1 µg/mL of doxycycline. Forty-two hours later, half of the cells were pulsed for 90 min with 10^−8^M S8L peptide. Finally H-2K^b^-S8L complexes were detected by sequential incubation with mAb 25D1.16 (10 µg/mL), FITC-labeled goat anti-mouse Abs (1∶50; Biolegend), and Alexa488-labeled goat anti-FITC Abs (1∶100; Invitrogen). Fluorescence was read on a FACSCalibur cytometer.

#### Cell line transfected with OVA

After a 36 Gy gamma-irradiation of cell lines, EG7 cells were diluted with EL4 cells while keeping a final number of 100,000 cells/well constant. One hundred thousand OT-I T cells labeled with 5 µM CarboxyFluorescein Succinimidyl Ester (CFSE; Invitrogen) for 12 min at 37°C were added to culture. After 20 h, supernatants were collected for quantification of IL-2 by ELISA whereas CFSE staining was measured after 48 h and 72 h using a BD FACSCAnto II cytometer (BD Biosciences) and FlowJo v10 software, and proliferation calculated as previously described [27]. For IL-2 ELISA, the capture anti-IL-2 mAb was clone JES6-1A12 and the detection mAb was clone JES6-5H4 (both BD Pharmingen), both used at 0.2 µg/50 µl/well. Incubation times were 2 h for supernatant, 45 min for detection mAb, and 30 min for streptavidin-HRP (Sigma). Tetramethylbenzidine was used as substrate.

### Ethics Statement

Animal experimentation performed in this study was approved by the Comité Régional d’Éthique pour l’Expérimentation Animale Ile de France – René Descartes (n° P2.LS.012.06).

### CD4+ T Lymphocyte Priming *in vitro and in vivo*

#### *In vivo* experiments

TCR-transgenic CD4+ T cells purified from skin-draining lymph nodes of BDC2.5/NOD mice were labeled with 10 µM CFSE for 12 min at 37°C and injected i.v. at 2×10^6^/mouse into sex-matched non-diabetic NOD IDE wt or ko recipient mice. Twenty-four hours later, the mice were anesthetized using Xylazine plus Ketamine and injected in the four footpads with 500 ng of an OVA fusion protein modified to carry the p31 mimotope (P3UOp31) that is specifically recognized by the BDC2.5 TCR, or with unmodified OVA fusion protein (P3UO). Prior to injection, the fusion proteins were non-covalently coupled to a mAb recognizing CD11c (clone N418; ATCC), as described previously [27]. Four days later, skin draining lymph nodes and pancreatic lymph nodes were purified and stained with rat anti-mouse CD4/Pacific Blue (clone RM4-5; BD Biosciences) and anti-mouse Vβ4/PE (clone KT4; BD Biosciences) Abs to identify the BDC2.5 cells. CFSE staining of BDC2.5 cells was measured using a FACSCFortessa™ flow cytometer (BD Biosciences) and FlowJo v10 software, and proliferation calculated as previously described [27].

#### *In vitro* experiments

Spleens from non-diabetic NOD IDE wt or ko were digested with collagenase D (Roche) for 40 min at 37°C and cell suspensions were enriched in DCs by performing a very low density gradient with OptiPrep (Axis-Shield); cells were stained for 30 min with anti-CD19/PE (clone 6D5; Biolegend), anti-CD11c/eFluor450 (clone N418; eBioscience) and 7-AAD was added prior cell sorting on a BD FACS ARIA-II cell sorter. Conventional DCs were sorted as 7-AAD-CD19-CD11c^hi^ cells. Fifty thousand cells were then incubated with serial dilutions of P3UOp31 or P3UO complexed with anti-CD11c as described above for 1 h before the addition of 50,000 TCR-transgenic CD4+ T cells purified from skin-draining lymph nodes of BDC2.5/NOD mice and labeled with 5 µM CFSE. On day 2, supernatants were collected to measure IL-2 concentration by ELISA, as described above. On day 4, proliferation of CD4+ T cells was evaluated by flow cytometry, as described above.

## Results

### Down-regulation of IDE Expression by siRNA Transfection of Human Cell Lines

**Figure 1:**
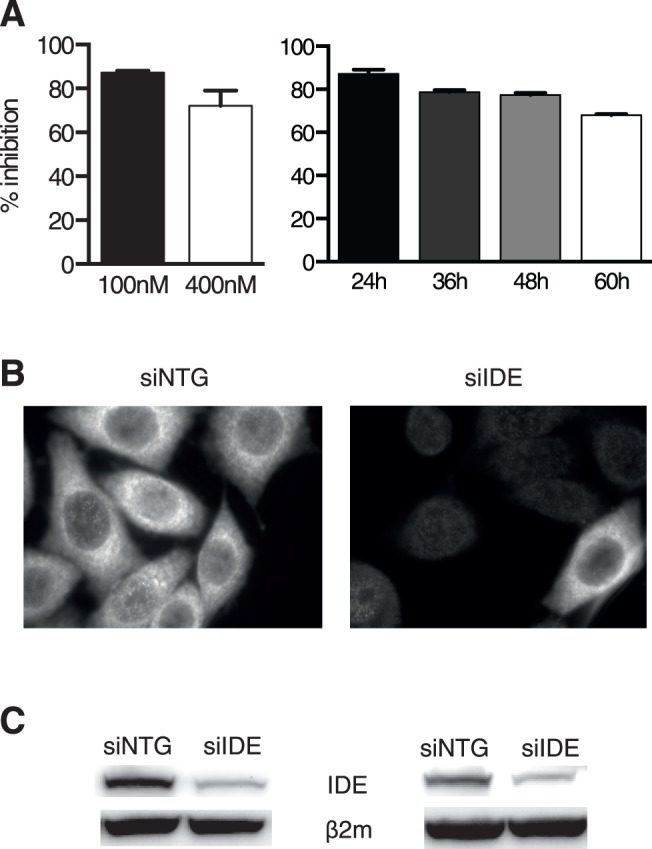
Efficiency of IDE down-regulation by siRNA. **A**, percentage of IDE mRNA inhibition, measured by qRT-PCR, in HeLa HHD cells transfected with different concentrations (4×100 or 4×400 nM) of specific siRNA (left panel), and at different time points after transfection by 4×100 nM siRNA (right panel). **B**, fluorescence microscopy analysis of HeLa HHD cells transfected with 400 nM of siIDE and siNTG 72 h after transfection. IDE expression was detected by staining with anti-IDE mAb 9B12. **C**, IDE expression by HeLa cells (left panel) and HEK293 cells (right panel) transfected with 4×100 nM of siIDE or siNTG and probed 48 h later by immunoblot. β_2-_m served as a loading control. siNTG, small interfering RNA, non-targeted; siIDE, small interfering RNA, IDE-specific; β_2-_m, beta 2-microglobulin. One out of 5 (**A, C**) and 2 (**B**) experiments is shown.

Because the previous observation of a role of IDE in class I antigen processing was made in a human cell line [18], we first set up protocols to reduce IDE expression in a selection of human cell lines. We electroporated cells with pools of 4 siRNA sequences and analyzed the effect on IDE expression using quantitative PCR (qPCR), fluorescence microscopy, and immunoblotting (Fig. 1). Expression of IDE mRNA was reduced by >80 percent at 24 h after transfection and then recovered slightly to ∼30 percent of controls at 60 h; optimal siRNA efficacy was observed at 100 nM duplex concentration, which was therefore used in further experimentation (Fig. 1A). Incomplete reduction of IDE expression as seen by qPCR likely was due to unaltered IDE expression in a small percentage of cells combined with complete extinction in the majority rather than uniform reduction in all cells, as suggested by microscopy analysis of HeLa cells (Fig. 1B). Efficient down-regulation of IDE expression was confirmed by immunoblots on HeLa and HEK293 cells analyzed 2 days after transfection (Fig. 1C). Knockdown protocols reducing IDE protein expression by at least 70 percent were established for all human cell lines used in this study, using the methods shown in Fig. 1.

### Effect of IDE Expression on Global Cell Surface HLA Class I Expression

**Figure 2:**
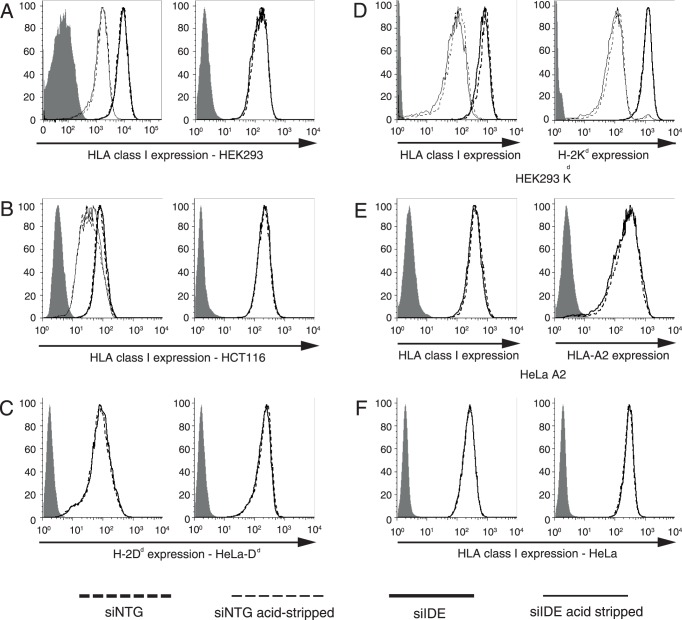
Effect of IDE knockdown on HLA class I expression. The indicated cell types were transfected with 4×100 nM siIDE and expression of MHC-I molecules was detected 48 h later by flow cytometry. In **A**, **B** and **D** (left panels) cells were acid-stripped 24 h prior to analysis. In **A**, **B**, **C**, **F** (right panels), cells were incubated with 400 U/ml of IFN-γ 24 h after transfection. Cell surface expression of “HLA class I” was assessed using mAb W6/32, while expression of H-2D^d^ was measured using mAb34-5-8, H-2K^d^ using mAb20-8-4 and HLA-A2 using mAb BB7.2. Filled histograms: secondary Ab only. One of 3 experiments is shown.

We next examined the effect of reduced IDE expression on the level of HLA-A, B, C molecules or of specific human or murine MHC-I allomorphs on the surface of human cell lines. IDE knockdown had no effect on steady state levels of total HLA class I molecules by HEK293, HCT116 and HeLa cells (Fig. 2A, B, F, left hand panels), including clones transfected by H-2K^d^ or HLA-A2 (Fig. 2D, E, left). Abs detecting the transfected class I molecules H-2D^d^, H-2K^d^ and HLA-A2 (Fig. 2C left and 2D, E right) also did not reveal an effect of the IDE expression levels on cell surface class I levels. Treatment with IFN-γ increases synthesis of MHC-I molecules but also may increase the number of oxidized proteins [28]. Reasoning that an effect of IDE knockdown might only be revealed in the presence of a high demand for class I ligands by newly synthesized class I molecules and/or damaged proteins, we treated cells with IFN-γ for 24 h, however again without noting any impact of IDE knockdown on HLA A, B, C expression by HEK293, HCT116 and HeLa cells (Fig. 2A, B, F right). Recovery of HLA class I expression, measured 24 h after acid stripping of cell surface molecules, also was not affected by IDE expression levels. Thus, IDE did not have any measurable impact on global HLA class I expression at all conditions tested.

### Effect of IDE on Antigen Presentation by MHC-I Molecules

**Figure 3:**
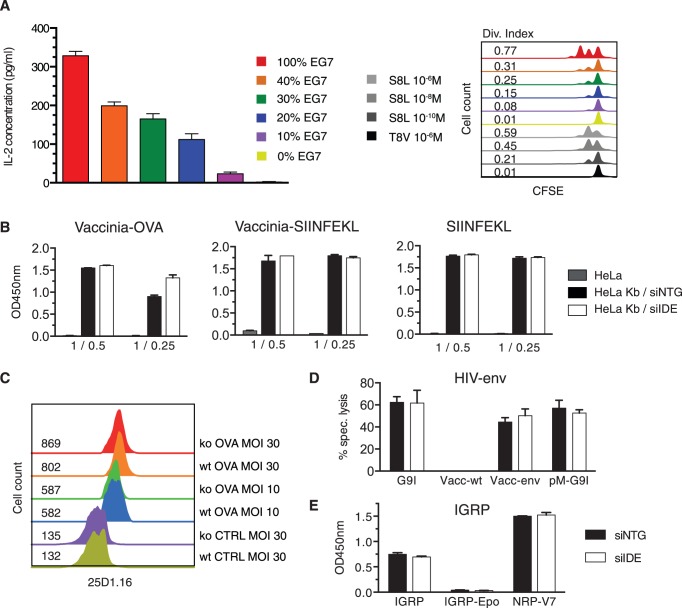
IDE is not involved in endogenous MHC-I presentation of viral antigens and a self antigen. A, 150,000 CFSE-labeled OT-I T cells were incubated with EG7 cells complemented to 100,000 cells with EL4 cells, with the percentage of EG7 cells in the mixture indicated in the legend. As control, 100,000 EL4 cells were incubated with S8L or irrelevant peptide TSYFESEV (T8V). After 16 h (left) and 48 h (right), T cell activation was assessed by measuring the IL-2 concentration in the supernatants and T cell proliferation by the dilution of CFSE, respectively. **B**, HeLa-K^b^ cells were transfected with siRNA. Seventy-two hours later, the cells were infected with a recombinant vaccinia virus encoding ovalbumin or the H-2K^b^ restricted epitope SIINFEKL, or pulsed with 10^−10^M SIINFEKL peptide and incubated with SIINFEKL-specific OT-I T cells, using different effector to target ratios. Secretion of IFN-γ by OT-I cells was measured by ELISA. In panel **C**, formation of S8L/H2-K^b^ complexes at the cell surface of IDE wt and ko C57BL/6 MEFs was evaluated 6 h after infection with vaccinia viruses expressing OVA or not (CTRL), by staining cells with mAb 25D1.16; numbers indicate the MFI for 25D1.16. Panel **D** shows an equivalent experiment with siRNA transfected HeLa-D^d^ cells and CTL recognizing a peptide from HIV gp160. Here the antigens were (from left to right) 10^−7^M cognate peptide G9I, wt vaccinia virus, vaccinia virus encoding HIV-env, and a plasmid encoding peptide G9I (pM-G9I). Presentation was assessed using a standard kill assay with an effector to target ratio of 2∶1. **E,** HEK293-K^d^ cells stably expressing an IGRP-GFP fusion protein were transfected with siRNA and acid stripped 56 h after transfection (IGRP). Prior to addition of CTLs at an E:T ratio of 1∶1, 72 h after transfection, part of the target cells was incubated with epoxomicin (Epo). Secretion of IFN-γ by CTLs was measured by standard ELISA. Cells treated as described and pulsed with 10^−6^M super-agonist peptide NRP-V7 were positive controls. One out of 3 (A, **B**, **D**), 2 (**C**) or 5 (**E**) experiments is shown.

Although IDE did not affect global cell surface MHC-I expression, it was possible that the enzyme affected presentation of individual epitopes produced in the endogenous MHC-I processing pathway. Because up to 30 percent of transfected cells retained IDE expression after siRNA transfection (Fig. 1), we first examined the sensitivity of our T cell assays. For this, we mixed transfected EG7 cells stably expressing OVA with non-transfected parent EL4 cells, keeping the total cell number constant, and incubated the mixture with OT-I CD8+ T cells that recognize the H-2K^b^-restricted epitope OVA_257-64_ (S8L). Using both IL-2 secretion and proliferation as readout, absence of OVA presentation by 60 percent or more of the cells was clearly detectable, ruling out a plateau effect for the highly sensitive OT-I T cells (Fig. 3A). Next we tested the effect of IDE knockdown on presentation of S8L and of two additional epitopes derived from the envelope protein of HIV and the type 1 diabetes autoantigen IGRP. The two former were expressed through recombinant vaccinia viruses and the latter in a stable transfectant. All epitopes were presented by murine class I molecules transfected in HeLa or HEK293 cells. Presentation of S8L pulsed on HeLa-K^b^ cells, expressed as mini-gene or as full-length protein, was not affected by IDE knockdown (Fig. 3B). Thus, cell surface expression of H-2K^b^, intracellular loading of K^b^ molecules, and proteolytic generation of the OVA epitope did not require IDE. To further corroborate the absence of an IDE effect on S8L presentation, we took advantage of the TCR-like mAb 25D1.16 [29] that provides quantitative information on the number of H-2K^b^ complexes. MEFs produced from published IDE-deficient C57BL/6 mice and infected with vaccinia viruses encoding OVA or not expressed the same number of H-2K^b^/S8L complexes as wild type MEFs (Fig. 3C), confirming that IDE was not involved in endogenous OVA processing for MHC-I presentation. Equivalent results were obtained when infecting BM-DCs (not shown). Similarly, presentation of the H-2D^d^-restricted HIV-envelope epitope encoded in a vaccinia virus or introduced by transfection with a plasmid encoding HIV-env was not affected by IDE knockdown (Fig. 3D). Finally, reduced IDE expression affected neither the presentation of a synthetic variant (NRP-V7) of the IGRP epitope nor the presentation of the epitope by a stable IGRP transfectant tested 16 hrs after removal of H-2K^d^-IGRP_206-14_ complexes by acid stripping, while treatment with the proteasome inhibitor epoxomicin abolished presentation (Fig. 3E). Thus, proteolytic generation of three viral and self-epitopes did not involve IDE.

### Effect of IDE knockdown on Cell Surface Expression of HLA-A3 and HLA-B27

**Figure 4:**
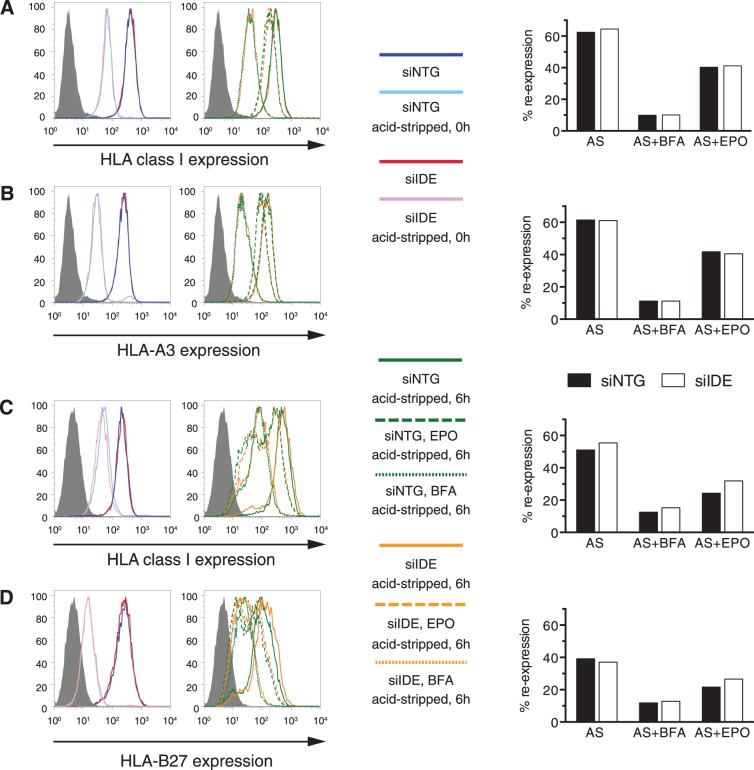
Role of IDE in expression of “proteasome-independent” HLA class I alleles. HLA-A3-expressing HEK293 cells and HLA-B27-transfected HeLa cells were transfected with 4×100 nM nM siRNA. Sixty-five hours later, 1 µM epoxomicin or 10 µg/ml brefeldin A were added to the cultures for 2 h or 30 min, respectively, before removal of most cell surface class I molecules by acid stripping and another 6 h incubation with the same drugs. HLA class I expression was detected using mAb W6/32 immediately after acid stripping (**A**, **C** left panel) and after the 6 h incubation (**A**, **C** center panel). The panels on the right show HLA class I re-expression relative to untreated cells as histograms. The panels in **B** and **D** show an evaluation of HEK293 cells using mAb GAP.A3 with specificity for HLA-A3 (**B**) and of HeLa-B27 cells using mAb ME-1 recognizing HLA-B27 (**D**). AS, acid stripping; BFA, brefeldin A; EPO, epoxomicin; filled histogram, secondary Ab only. One out of 3 experiments is shown.

The effect of proteasome inhibition on global MHC-I loading has been described to vary greatly according to the allomorph considered. The relative insensitivity of HLA-A3 to proteasome inhibitors is ascribed to its preference for Lys at the C-terminus [10], while the reason for the reported proteasome resistance of a 30 percent proportion of HLA-B27 ligands, most of which are derived from small basic proteins, remains unclear [11]. Although we had found that expression of the HLA and H-2 class I molecules expressed by HeLa, HEK and HCT cells did not correlate with IDE expression levels, it was conceivable that IDE was involved in ligand production for the mentioned particularly “proteasome-independent” allomorphs. To study this, we subjected HEK293 cells naturally expressing HLA-A3, and HLA-B27-transfected HeLa cells to acid stripping of cell surface class I molecules, and examined re-expression of total HLA-A, B, C molecules versus re-expression of the presumed proteasome-independent or partly independent molecules. Six hours after stripping, global class I and A3 re-expression reached 50 to 60 percent of levels prior to acid treatment (Fig. 4A–C), while B27 re-expression was 40 percent (Fig. 4D). Incubation with Brefeldin A after acid-stripping reduced re-expression to 10 percent, as expected. Addition of epoxomicin after stripping reduced re-expression by no more than one third to one half, consistent with the reported proteasome-independence of the allomorphs studied [8], [10], [11]. Note that the epoxomicin sensitivity of HLA-A3 did not differ from that of the HLA-A,B,C molecules expressed by HEK293, while HLA-B27 appeared more “proteasome independent” than the endogenous HLA-A,B,C molecules expressed by HeLa cells. Knockdown of IDE had no effect on the recovery of HLA-A3 and HLA-B27 (Fig. 4). Thus, IDE is not responsible for the reported “proteasome independence” of HLA-A3 and HLA-B27.

### Effect of IDE Knockdown on Presentation of Amyloid Beta and Proinsulin

**Figure 5:**
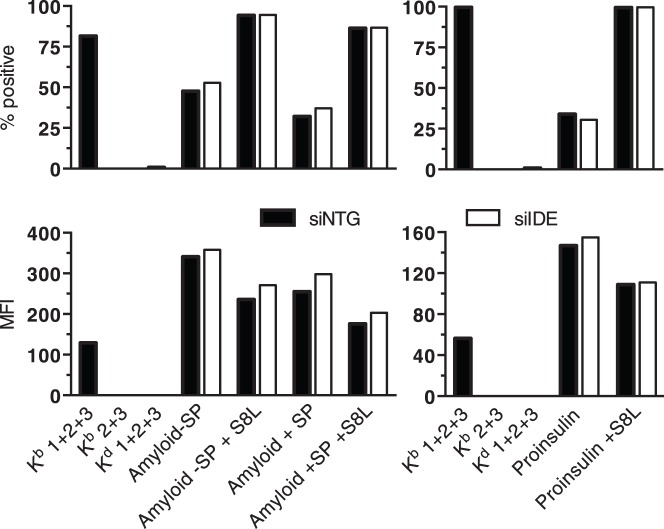
HeLa Tet-on cells expressing H-2K^b^ were nucleofected with 4×100 nM siRNA. Twenty-four hours later, the cells were electroporated with inducible plasmids encoding amyloid beta preceded by a signal peptide (+SP) or not (−SP), or encoding proinsulin, all tagged with the S8L peptide at the C-terminus. Protein expression was induced immediately by addition of 1 µg/ml doxycylin. Forty-eight hours later, K^b^/S8L complexes on the cell surface were detected using mAb 25D1.16 (Ab1) followed by FITC-labeled goat anti-mouse Abs (Ab2) and Alexa488-labeled goat anti-FITC Ab (Ab3). Control samples were HeLa-K^b^ cells pulsed for 2 h with 10^−8 ^M S8L and stained with Abs 1, 2 and 3 or with Abs 2 and 3 only, as well as peptide-pulsed HeLa cells expressing H-2K^d^ stained with Abs 1, 2 and 3. One of two experiments is shown.

Although IDE played no role in class I presentation of 3 standard epitopes (Fig. 3), we reasoned that a role was more likely in presentation of substrates known to be degraded by IDE and to be relatively resistant to other proteases. Only a small number of proteases can degrade beta amyloid [30] and proinsulin, though having lower affinity for IDE than insulin, is known to be an IDE substrate [31]. Lacking suitable CD8+ T cells recognizing beta amyloid and proinsulin, we tagged the two autoantigens with the epitope S8L, expressed them in H-2K^b^-transfected HeLa cells, and measured presentation using mAb 25D1.16 Expression of both beta amyloid (preceded or not by a signal peptide) and proinsulin resulted in presentation of S8L by 30 to 50 percent of transfected cells, suggesting that both proteins were efficiently processed (Fig. 5). However, IDE knockdown did not reduce presentation.

### Analysis of IDE Deficient Mice for Expression of MHC Molecules and MHC Class II Antigen Presentation

**Figure 6:**
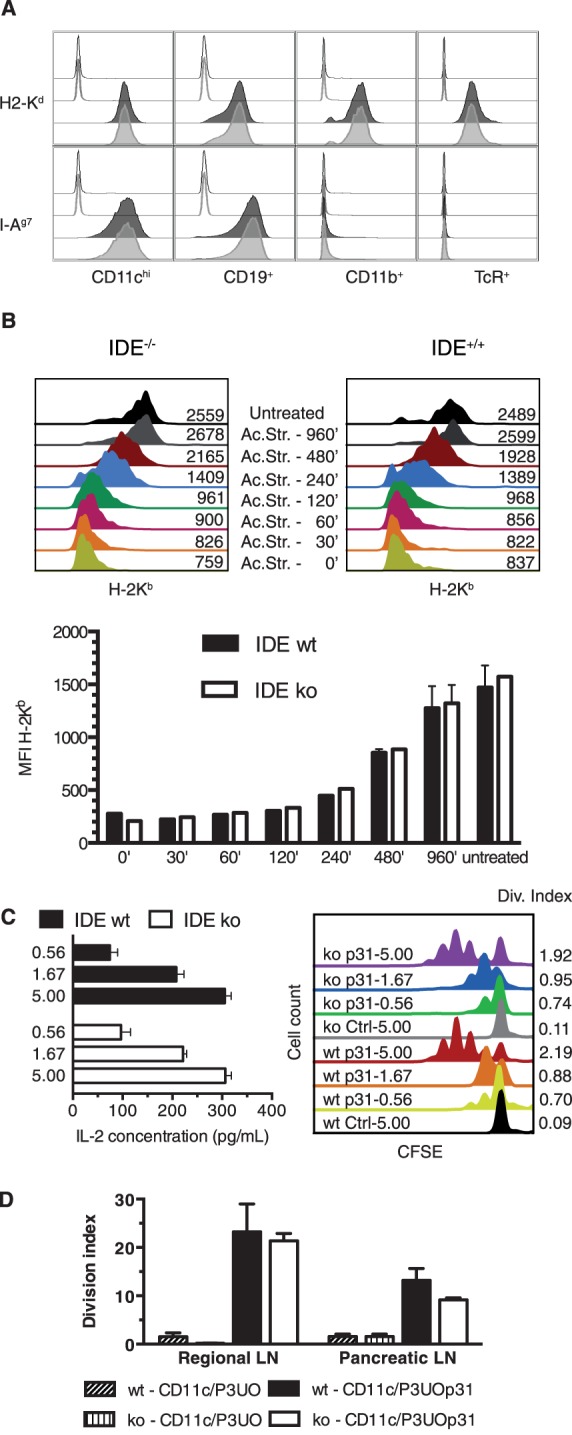
Characterization of IDE ko mice. **A**, expression of MHC molecules on wt (black lines) and IDE ko (grey lines) NOD splenocytes. Live cells were gated on TcR-β+ cells, CD19+ cells, CD11c^hi^ cells or CD11b+CD11c− cells, and analyzed for expression of H-2K^d^ and I-A^g7^. Isotype controls are shown in open, and specific stainings in filled histograms. **B**, Splenocytes from IDE wt and ko C57BL/6 mice were subjected to a 90 s treatment with acid, incubated with 10% FCS complete medium for the indicated periods to allow for re-expression of MHC-I molecules, and then stained for H2-K^b^ with mAb AF6-88.5, using gates on CD11c+ DCs (top panels, experiment represented as FACS plots) and CD19+ B cells (bottom panel, means ± SDEV of 2 experiments represented as histogram). Numbers on histograms in the top panels indicate MFI for AF6-88.5. **C**, 25,000 TCR-transgenic CFSE-labeled BDC2.5 CD4+ T cells were put in contact with 20,000 sorted splenic CD11c^hi^ DCs incubated with graded amounts of the fusion proteins P3UOp31 or P3UO (Ctrl) in complexes with an anti-CD11c mAb. After 2 days (left panel) or 4 days (right panel), T cell activation was assessed by measuring the IL-2 concentration in the culture supernatant and proliferation was measured by flow cytometry as dilution of CFSE, respectively. The numbers next to the FACS plots indicate the division index of progenitor cells, calculated as previously described [27]. One out of 2 experiments. **D**, priming of CD4^+^ T cells in IDE-deficient mice. CFSE-labeled BDC2.5 T cells were injected into wt and IDE-deficient mice. Twenty-four hours later, 500 ng of the P3UOp31 or P3UO fusion proteins in complexes with a CD11c-specific mAb were injected s.c. Four days after antigen injection, CD4^+^/Vβ4^+^ T cells recovered from draining and pancreatic lymph nodes were assessed for CFSE dilution by flow cytometry and the division index of injected precursors was calculated. Two independent experiments were performed, with a total of 5 or 6 mice per condition. Means and SDEV are shown.

Although IDE has a dominant cytosolic location, a small percentage of the enzyme is thought to localize to endosomes and may participate in the degradation of internalized insulin according to some older papers [31]. To further address a possible role of IDE in production of MHC-I ligands, and to examine its role in production of MHC-II ligands, we used the published IDE knockout mice generated on the C57BL/6 background, which we also back-crossed to the NOD strain (AM and PVE, manuscript in preparation). IDE–deficient mice on both genetic backgrounds harbor normal numbers of splenic B, T and dendritic cells which express normal amounts of MHC-I molecules, confirming results obtained with human cell lines using RNA interference (Fig. 6A, Table 1). We reasoned that, although the steady state levels of MHC-I molecules on IDE ko cells are normal, a potential limited role of IDE in endogenous antigen processing might cause a delay in production and export of new class I molecules. To address this, we subjected IDE wt and ko C57BL/6 splenic DCs and B lymphocytes to a short acid treatment that removed 85% of cell surface H-2K^b^ molecules and monitored re-expression of H-2K^b^ over up to 16 h (Fig. 6B). IDE ko cells recovered class I expression with the same kinetics as wt cells. Similar results were obtained with NOD splenocytes (not shown).

**Table 1:** MHC-I expression by splenocyte sub-populations of IDE ko mice.

Strain	MFI* control	% B cells	MFI	% T cells	MFI	% DCs	MFI
C57/BL6 wt	3.0	64	93	31	93	5.9	90
C57/BL6 ko	−	64	114	31	113	5.0	120
NOD wt	3.7	46	146	17	132	4.2	166
NOD ko	−	48	150	17	124	4.0	138

*MFI, mean fluorescence intensity.

DCs, B and T cells and macrophages of IDE ko NOD mice also expressed normal levels of cell surface MHC-II molecules (Fig. 6A). Importantly, the murine cell types tested by us for MHC-I and II expression all physiologically express IDE (between 3,000 and 7,000 IDE mRNA copies per cell; J. Kim and PvE, unpublished observations), consistent with the known ubiquitous nature of its expression. To study MHC-II-restricted antigen presentation, we used complexes of a fusion protein consisting of OVA preceded by streptococcal Ig-binding domains and ubiquitin, and a mAb with specificity for the dendritic cell receptor CD11c [27]. Two versions of the fusion protein were used: protein P3UOp31 containing the p31 mimotope [32] inserted into OVA and recognized by TCR-transgenic CD4^+^ BDC2.5 T cells (specific for the pancreatic autoantigen chromogranin A [33]), and protein P3UO containing unmodified OVA. Upon incubation with graded amounts of fusion protein *in vitro*, IDE-sufficient and deficient NOD DCs induced IL-2 secretion by, and proliferation of BDC2.5 CD4+ T cells with undistinguishable efficiency (Fig. 6C). We also injected mice with CFSE-labeled BDC2.5 cells followed 1 day later by the antigenic complexes, and another 4 days later by analysis of T cell proliferation (Fig. 6B). While the control protein P3UO did not stimulate BDC2.5 proliferation, injection of CD11c-targeted P3UOp31 triggered vigorous proliferation of BDC2.5 cells recovered both from regional and from pancreatic lymph nodes. However, IDE deficiency did not have a significant impact on the extent of BDC2.5 proliferation.

## Discussion

Prompted by the published finding that IDE can process a tumor antigen [18], in this study we examined the hypothesis that proteolysis by IDE may account for the phenomenon of proteasome-independent MHC-I loading with peptides, suggested by various reports in the literature. Studying human and murine cells, and using both RNA interference and gene invalidation, we conclude that IDE neither has a detectable effect on cell surface expression of various MHC-I molecules including allomorphs described to be especially proteasome-independent, nor on presentation of five different antigens including two proteins known to be preferred IDE substrates. Thus, implication of IDE in MHC-I processing as described for MAGE-A3 is unlikely to be a common phenomenon.

Given the number of MHC-I allomorphs studied and the different approaches used in this study, we conclude that a general major role of IDE in MHC-I loading can be ruled out. Our findings therefore parallel earlier studies of TPPII, the second protease initially described to produce an antigenic epitope in a proteasome-independent manner and subsequently shown to be dispensable for efficient MHC-I loading. Thus, despite substantial efforts undertaken in this and previous studies, it remains impossible to identify a protease capable of making a significant proteasome-independent contribution to cytosolic MHC-I antigen processing. One way, and possibly the most plausible one, of interpreting this fact is that such a protease does not exist, and that previous reports of substantial proteasome-independent MHC-I loading simply reflect the conundrum that complete proteasome inhibition (and more so knockout) of the proteasome is not feasible, and that some class I allomorphs are more readily loaded in the presence of low proteasome activity than others. It also cannot be ruled out that some allomorphs rely more on non-cytosolic sources of ligands than others.

Our examination of the presentation of defined epitopes by cells lacking IDE demonstrated that the enzyme is not implicated in presentation of three standard epitopes as well as two epitopes linked to proteins known to be preferred IDE substrates. Although these results are consistent with the lack of a global role of IDE in antigen processing, they do not rule out a role of the enzyme in processing and presentation of peptides derived from preferred substrates. Lacking CD8+ T cells recognizing beta amyloid, we attached an OVA epitope to the protein and examined its presentation to OT-I T cells. It is conceivable that other proteases, e.g. the proteasome, can remove the C-terminal tag from our beta amyloid protein without degrading the amyloid 42-mer. However, our results obtained in an independent project suggest that IDE is not required for processing and presentation of an immunodominant epitope in the insulin B chain by beta cells (AM and PVE, manuscript in preparation), confirming the results obtained with the tagged insulin molecule examined in this study. Nevertheless, it needs to be emphasized that our results do not rule out an effect of IDE in processing of other antigens, or minor effects on presentation of the epitopes studied here. On the other hand, the absence of other “accessory” proteases involved in MHC-I antigen processing, such as ERAP1 or immunoproteasome subunits, consistently results in decreased cell surface expression of class I molecules [34], [35], suggesting that the extensive screening of different class I allomorphs undertaken in this study should have allowed for detecting a global role of IDE in antigen processing.

Although the negative results reported here might seem little surprising as they are in line with earlier studies of other proteases, we contend that the complete lack of a role of IDE in substrates such as amyloid beta and insulin is a non-trivial observation. Few proteases including IDE but not the proteasome are able to degrade beta amyloid efficiently [30]. Moreover, although the proteasome is known to cleave the insulin B chain efficiently, insulin and proinsulin are highly preferred IDE substrates [31]. IDE also has been demonstrated to degrade proinsulin processed by the endoplasmic reticulum-associated degradation pathway [36]. Thus a role for IDE in the processing of at least these substrates was expected. We speculate that these proteins may not be readily accessible to IDE when they are processed to antigenic epitopes, consistent with the so far entirely hypothetical model of an “immunoribosome” [37] including a co-translational mechanism for peptide degradation in which the proteasome would occupy a privileged and exclusive place.
